# Genome-wide analysis of the sox family in the calcareous sponge *Sycon ciliatum*: multiple genes with unique expression patterns

**DOI:** 10.1186/2041-9139-3-14

**Published:** 2012-07-23

**Authors:** Sofia Fortunato, Marcin Adamski, Brith Bergum, Corina Guder, Signe Jordal, Sven Leininger, Christin Zwafink, Hans Tore Rapp, Maja Adamska

**Affiliations:** 1 Sars International Centre for Marine Molecular Biology, Thormøhlensgt. 55, Bergen 5008, Norway; 2 Department of Biology and Centre for Geobiology, University of Bergen, Thormøhlensgt. 55, Bergen, 5008, Norway

## Abstract

**Background:**

Sox genes are HMG-domain containing transcription factors with important roles in developmental processes in animals; many of them appear to have conserved functions among eumetazoans. Demosponges have fewer Sox genes than eumetazoans, but their roles remain unclear. The aim of this study is to gain insight into the early evolutionary history of the Sox gene family by identification and expression analysis of Sox genes in the calcareous sponge *Sycon ciliatum*.

**Methods:**

Calcaronean Sox related sequences were retrieved by searching recently generated genomic and transcriptome sequence resources and analyzed using variety of phylogenetic methods and identification of conserved motifs. Expression was studied by whole mount in situ hybridization.

**Results:**

We have identified seven Sox genes and four Sox-related genes in the complete genome of *Sycon ciliatum*. Phylogenetic and conserved motif analyses showed that five of *Sycon* Sox genes represent groups B, C, E, and F present in cnidarians and bilaterians. Two additional genes are classified as Sox genes but cannot be assigned to specific subfamilies, and four genes are more similar to Sox genes than to other HMG-containing genes. Thus, the repertoire of Sox genes is larger in this representative of calcareous sponges than in the demosponge *Amphimedon queenslandica*. It remains unclear whether this is due to the expansion of the gene family in *Sycon* or a secondary reduction in the *Amphimedon* genome. *In situ* hybridization of *Sycon* Sox genes revealed a variety of expression patterns during embryogenesis and in specific cell types of adult sponges.

**Conclusions:**

In this study, we describe a large family of Sox genes in *Sycon ciliatum* with dynamic expression patterns, indicating that Sox genes are regulators in development and cell type determination in sponges, as observed in higher animals. The revealed differences between demosponge and calcisponge Sox genes repertoire highlight the need to utilize models representing different sponge lineages to describe sponge development, a prerequisite for deciphering evolution of metazoan developmental mechanisms.

## Background

The Sox genes (Sry related high mobility group, HMG box) are a family of transcription factors with important roles in regulating development and cell fate determination throughout the animal kingdom [1,2]. The Sox proteins are characterized by the HMG DNA binding domain of 79 amino acids, resembling the mammalian testis determination factor, Sry, which was the first Sox domain identified [3]. There are 20 Sox genes in mammals [4] which have been classified in five groups of Sox proteins (B, C, D, E, and F) [5]. However, additional groups have been created to accommodate divergent genes with limited taxonomic distribution, for instance group J [5]. Groups B, C, E, and F are found in all eumetazoan lineages, but group D is found only in the bilaterians [5].

No Sox genes are present in the sequenced genomes of the unicellular choanoflagellate, *Monosiga brevicollis*[6], or the amoeboid holozoan *Capsaspora owczarzaki*[7]. Since they are present in basal metazoans like sponges (that is, the demosponge *Amphimedon queenslandica*) [8,9] and placozoans (*Trichoplax adhaerens*) [10], they have likely arisen in the last common ancestor to the Metazoa [8]. There is a larger repertoire of Sox genes in cnidarians [11-13] and the ctenophore *Pleurobrachia pileus*[14] than in the demosponges [8,9,15] and the placozoans [10]. Previous phylogenetic analysis of cnidarian Sox genes including the species *Hydra magnipapillata*, *Nematostella vectensis*, and *Clytia hemisphaerica* placed some of these sequences into the previously identified groups of Sox genes; however some of these genes cannot be classified into any specific group [11-13]. The expression patterns of cnidarian Sox genes suggest that they have roles in a wide variety of developmental functions, such as germ layer formation, organ development, cell type specification, and neural development [11-13].

Previous studies on Sox genes in sponges include the two demosponges, *Amphimedon queenslandica*[8,9] and *Ephydatia muelleri*, as well as the calcareous sponge *Sycon raphanus*[15]. In *Amphimedon*, four Sox genes have been found, including two members of group B (*AmqSoxB1* and *AmqSoxB2*) and single members of groups C and F [9]. Sox genes from *Ephydatia* and *Sycon raphanus* could not be clearly classified due to incomplete domain sequences included in the phylogenetic analyses [15]. As a consequence, the complement of Sox genes in calcareous sponges is still unclear. In addition, apart from an RT-PCR study suggesting dynamic expression of Sox genes during embryonic development in *Amphimedon*[9], no expression patterns on a cellular level are published for this or any other sponge. For this reason, more studies in sponges are required to fully understand the function of Sox genes in the phylum Porifera in comparison with the Eumetazoa. The aims of this study were to analyze the repertoire of Sox genes in the calcareous sponge *Sycon ciliatum* and to trace their expression during development.

**Figure 1:**
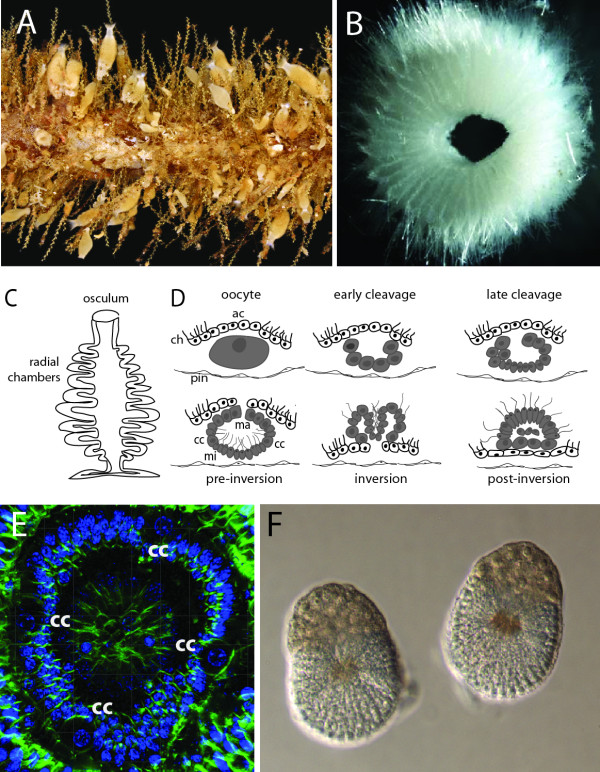
***Sycon ciliatum: *morphology and embryonic development.** (**A**) Environmental sample of multiple specimens of *Sycon* growing on stipe of the kelp *Laminaria hyperborea*. (**B**) Transverse section of *Sycon ciliatum* demonstrating radial symmetry. (**C**) Schematic representation of *Sycon* body plan. (**D**) Schematic representation of key stages in embryogenesis (after [17]): top; oocyte, early and late cleavage stage; bottom, pre-inversion stage, inversion and post-inversion. (**E**) Confocal image of an embryo during pre-inversion stage showing four cruciform cells (cc) among micromeres. Actin cytoskeleton is labeled green, DNA is blue. (**F**) Larvae. Cell types are abbreviated as follows: ac, accessory cells; cc, cruciform cells; ch, choanocytes; ma, macromeres; mi, micromeres; pin, pinacocytes.

*Sycon ciliatum* is an attractive model system for developmental biology studies [16]. This sponge is a common and abundant (Figure 1A) species found in shallow waters in the North-East Atlantic, with a distribution extending from The Channel in the south to Svalbard and Greenland in the north (Rapp, unpublished work). It has the typical body plan of syconoid sponges where choanocyte-lined radial chambers surround an endopinacocyte-lined atrial cavity leading to a single osculum; the outer surface of the body is covered by exopinacocytes (Figure 1B, C). Embryogenesis of *Sycon ciliatum* and related species has been well studied [17-19] and it takes place in the mesohyl, a narrow space located between the pinacocytes and choanocytes (Figure 1D). Symmetric cleavage followed by cell differentiation leads to formation of a cup-shaped embryo composed of numerous ciliated micromeres, a lower number of larger macromeres, and four cruciform cells symmetrically distributed among the micromeres (Figure 1D, E). The embryo undergoes inversion while it translocates to the radial chamber (Figure 1D), and the mature larva (Figure 1F) swims through the oscular opening. Both larva and adult display clear single body axes; the larva has unique tetra-radial symmetry (conferred by the cruciform cells) while the adult is radially symmetrical. 

Recently generated complete draft genomic sequence and extensive transcriptome resources allow us to perform whole-genome analysis of developmentally important gene families (Adamski *et al.*, unpublished work), and established *in situ* hybridization protocols allow for studies of gene expression in all life stages.

## Results

### Phylogenetic analysis of *sycon* Sox genes

We have identified 12 HMG domains corresponding to 11 *Sycon ciliatum* Sox- related genes within the genomic and transcriptomic resources generated by a combination of traditional and next generation sequencing (Table 1).

**Table 1:** ***Sycon *and *Leucosolenia *Sox genes**

**Species**	**Seq. no**	**Internal ID number of retrieved sequence**	**First hit on NCBI BlastX search**	**Accession number**	**E value**	**Name given after phylogenetic analysis**	**Accession number*Sycon*and*Leucosolenia***
*Sycon*	1	Sci475726	SoxpB *Acropora millepora*	ABD97869	5e^-25^	*SciSoxB*	JX171144
*ciliatum*	2	Sci445174	Sox21B *Danio rerio*	AAS47833	2e^-15^	*SciSoxC*	JX171145
	3	Sci447413	Sox8 *Oncorhynchus keta*	AAV38119	4e^-25^	*SciSoxE*	JX171146
	4	Sci115371	Sox18 *Xenopus Silurana*	AAI67402	9e^-14^	*SciSoxF1*	JX171147
	5	Sci56754	HMG box *Brugia malayi*	EDP37253	7e^-25^	*SciSoxF2*	JX171148
	6	Sci22777	Sox8 *Gallus gallus*	AF228664	3e^-22^	*SciSox6*	JX171149
	7	Sci95797	Sox17 *Homo sapiens*	NP_071899	1e^-18^	*SciSox7*	JX171143
	8	Sci63714	HMG *Brugia malayi*	EDP37253	5e^-12^	*SciSoxL1*	JX171150
	9	Sci115540	Sox13 *Takifugu rubripes*	AAQ18513	6e^-11^	*SciSoxL2*	JX171151
	10	Sci500969	Syr-box 32 *Oreochromis niloticus*	ABG11758	9e^-11^	*SciSoxL3*	JX171152
	11	Sci180533	Sox8 *Homo sapiens*	NP_055402	8e^-19^	*SciSoxL4a/b*	JX171153
*Leucosolenia*	1	Lco315339	Sox14 *Danio rerio*	XP_685850	2e^-26^	*LcoSoxB*	JX171154
*complicata*	2	Lco183	Syr 9 *Monodelphis domestica*	ACZ54381	5e^-25^	*LcoSoxE*	JX171155
	3	Lco136843	SoxF *Acropora millepora*	ACF33143	5e^-20^	*LcoSoxF*	JX171156
	4	Lco244	SoxBb *Acropora millepora*	ACF33140	1e^-22^	*LcoSox6*	JX171157
	5	Lco554456	SoxF *Lethenteron camtschaticum*	BAH58895	2e^-23^	*LcoSoxF2*	JX171160
	6	Lco122678	Sox13 *Ixodes scapularis*	EEC19583	3e^-11^	*LcoSoxL1*	JX171158
	7	Lco38077	Sox similar protein *Suberites domunluca*	CBK62691	4e^-16^	*LcoSoxL4a/b*	JX171159

**Table 2:** **Summary of *Sycon *Sox and SoxL genes expression**

**Gene**	**Expression**	
***SciSoxB***	Oocytes, cleavage stage embryos, macromeres, and cruciform cells	
***SciSoxC***	Oocytes, cleavage stage embryos, macromeres	
***SciSoxE***	Choanocytes and some mesohyl cells	
***SciSoxF1***	Choanocytes and accessory cells, some mesohyl cells	
***SciSoxF2***	Large spindle-shaped cells around osculum	
***SciSox6***	Choanocytes, pinacocytes, small cells around osculum	
***SciSox7***	Ubiquitous during embryogenesis, choanocytes	
***SciSoxL1***	Oocytes, cleavage stage embryos, cruciform cells, choanocytes	
***SciSoxL2***	Oocytes, cleavage stage embryos, macromeres, choanocytes, small cells around osculum	

Dark grey indicates where the expression is detected; light grey indicates embryonic cells where expression is not detected; white represents non-embryonic cells. ac, accessory cells; cc, cruciform cells; ma, macromeres; mes, mesohyl cells; mi, micromeres.

We have performed phylogenetic analyses of HMG domain sequences of Sox genes using different combination of taxa and the 12 sequences of *Sycon* (data not shown). In the initial phylogenetic analysis, most of *Sycon* Sox genes did not clearly fall into the recognized Sox groups (data not shown). To test whether adding sequences from another sponge closely related to *Sycon* would help to resolve the phylogenetic tree, we additionally identified and included sequences of Sox genes from another calcareous sponge, *Leucosolenia complicata* (Adamski *et al.*, unpublished work). Up to date, we have recovered a total of seven Sox and Sox-related sequences from *Leucosolenia* (Table 1).

Another phylogenetic tree was then constructed including the entire repertoire of identified *Sycon* and *Leucosolenia* Sox genes (Additional file 1). However, this phylogenetic analysis also resulted in a non-resolved tree with multiple long-branch attraction artefacts [20]. To reduce long-branch attraction, the most divergent sequences from both *Sycon* and *Leucosolenia* were excluded from further analyses. The excluded genes resemble Sox genes but have a divergent conserved motif within the HMG domain: either at the motif RPMNAF (positions 5 to 10), and/or at YK/R (positions 70 to 72); we named them Sox-like genes: *SciSoxL1* to *SciSoxL4a/b* and *LcoSoxL1*, *LcoSoxL4a/b* (Table 1, Additional file 2).

**Figure 2:**
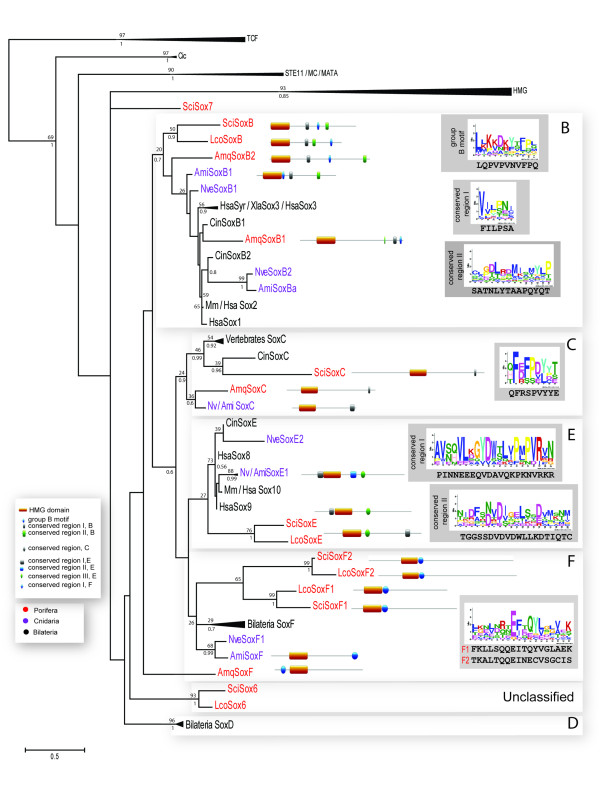
**Phylogenetic analysis of *Sycon *Sox genes based on the HMG domain sequences and schematic representation of motif conservation within groups B, C, E, and F.** Maximum likelihood tree using LG + G model of protein evolution is shown. Support values of posterior probabilities (bottom) and bootstrap (top) are displayed, BT values below than 10% and PP values below 0.5 were discarded. A root was placed in the out-groups. *P* values for *Sycon* motifs: Group B motif, 9.30E-07; conserved region I, 1.16E-04; conserved region II, 1.99E-05; Group C conserved region, 2.26E-07; Group E conserved region I, 9.03E-10; conserved region II, 4.83E-13; Group F conserved region, 1.40E-14 (*SciSoxF1*) and 1.10E-10 (*SciSoxF2*). Ami, *Acropora millepora*; Amq, *Amphimedon queenslandica*; Ce, *Caenorhabditis elegans*; Cin, *Ciona intestinalis*; Hsa, *Homo sapiens*; Lco, *Leucosolenia complicata*; Nv, *Nematostella vectensis*; Sci, *Sycon ciliatum.*

Overall, the final phylogenetic analyses of the non-divergent set of sequences shown on Figure 2 (see Additional file 3 for alignment) resolved most of *Sycon* Sox genes within the known Sox gene groups. The analysis also resolved Sox genes from Bilateria and Cnidaria within SoxB, C, D, E, and F groups supporting previous analyses [5,8,9,11-15]. However, SoxB group did not show a clear division into SoxB1 or SoxB2 clades. Five *Sycon* HMG domains of Sox genes can be assigned to the known eumetazoan Sox groups B, C, E, and F (Figure 2). Although the list of Sox genes in *Leucosolenia* might still be incomplete, so far all of the identified sequences have clustered with *Sycon* sequences. A SoxC gene in *Leucosolenia* has not been identified; this may be due to incomplete sequence resources for this species or represent genuine gene loss in *Leucosolenia*. In addition, our analysis suggests that an expansion of SoxF genes have occurred in the calcaronean sponges; we named these genes SoxF1 and SoxF2 (Table 1). 

Notably, our analysis did not reveal orthologous relationships between *Amphimedon* and calcaronean sequences even in cases where members of the same subfamily are present in both sponges, such as SoxB or SoxC. As reported by Larroux and colleagues [9] the *Amphimedon* SoxF gene did not cluster with other SoxF sequences in the maximum likelihood analysis. However, conserved motif analysis (see below) indicates that this gene belongs to the SoxF subfamily.

The remaining two *Sycon* Sox genes named *SciSox6* and *SciSox7* (Table 1) did not fall into any known Sox group, while clustering within the Sox family (Figure 2). One ortholog of *SciSox6* was found in *Leucosolenia,* and it was named *LcoSox6.* In contrast, we have not found a counterpart of *SciSox7* in *Leucosolenia*.

### Motif conservation within sponge Sox genes

We compared full length Sox proteins from *Sycon*, *Leucosolenia*, and *Amphimedon* with their homologs from different taxa (Figure 2, Additional file 4) to find conserved motifs outside the HMG domain. The analysis revealed the presence of a number of motifs that are conserved between the eumetazoan and poriferan sequences. However, the motifs in sponge sequences were often quite divergent as compared to their bilaterian and cnidarian counterparts (Figure 2, Additional file 4). *Amphimedon* SoxB1 and B2, *Leucosolenia* SoxB, and *Sycon* SoxB genes contained the B-group specific motif. In contrast to the eumetazoan SoxB proteins, the B-group specific motif in sponges was not located directly next to the C-terminal of the HMG domain, but appeared in different positions within the C-terminal part of the protein. Both *Amphimedon* and *Sycon* SoxC proteins contained a slightly divergent C-group motif as compared to *Homo* and *Acropora* SoxC. Two conserved regions were found for the *Sycon* SoxE protein while only one region was found in *Leucosolenia* SoxE. Finally, the conserved short SoxF motif was also found in the three sponge proteins, but was located closer to the HMG domain, while in *Acropora* and *Homo* it is located at the C-terminal of the protein.

### Sox genes are dynamically expressed during embryogenesis and cell differentiation

We have studied expression of Sox genes in adult sponges containing a wide variety of embryonic stages by whole mount *in situ* hybridization. Except for *SciSoxL3* and *SciSoxL4a/b*, for which we could not amplify probes suggesting they are not significantly expressed in adult cells or during embryogenesis, all other genes displayed unique patterns during development and/or in adult cells.

**Figure 3:**
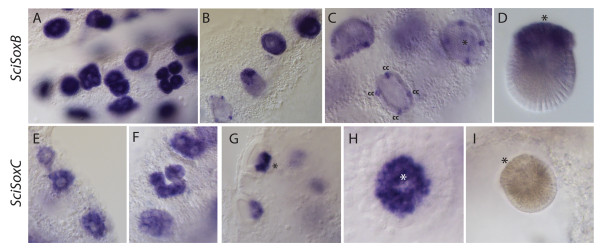
**Expression of *SciSoxB *and *SciSoxC *during embryogenesis.***SciSoxB* is strongly expressed in oocytes and during cleavage (**A**); the expression gradually decreases from the late cleavage (**B**, top right) to pre-inversion (B, bottom left), becoming limited to the cruciform cells (cc) in pre-inversion stage embryos (B and **C**) and macromeres (asterisk) of pre-inversion stage embryos (C) and larvae (**D**). *SciSoxC* is expressed in oocytes (**E**) and during cleavage (**F**); in pre-inversion stage embryos expression is limited to macromeres (asterisk) (**G**, **H**) and becomes undetectable in the larvae (**I**). All images are of glycerol-cleared slices of sponges containing developmental stages, except of D, demonstrating an isolated larva.

The expression of *SciSoxB* was strong in the oocytes and in blastomeres of early cleavage stages (Figure 3A). During pre-inversion it is specifically detected in macromeres and in the cruciform cells (Figure 3B, C). This pattern continues until early post-inversion, but when the larva is fully developed no expression can be detected in the cruciform cells (Figure 3D). *SciSoxC* was also detected in oocytes and in all blastomeres during cleavage (Figure 3E, F) but became restricted to macromeres during pre-inversion (Figure 3G, H). This expression becomes undetectable after inversion and in the larva (Figure 3I).

**Figure 4:**
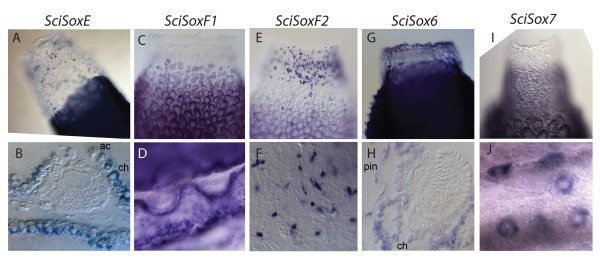
**Expression of *SciSoxE *,*SciSoxF1 *,*SciSoxF2 *,*SciSox6 *, and *SciSox7 *.***SciSoxE* is expressed in choanocytes (ch) (**A**, **B**) and a fraction of mesohyle cells, particularly prominent in the apical (oscular) part (A). The expression is undetectable in the oocytes and accessory cells (ac) (B). *SciSoxF1* expression is limited to choanocytes (**C**, **D**) and accessory cells (D), and absent in the embryos (D). *SciSoxF2* is weakly expressed in the choanocytes (**E**) and strongly in large spindle shape cells surrounding the osculum (E, magnified on **F**). *SciSox6* is strongly expressed in choanocytes (ch), pinacocytes (pin), and a variety of mesohyle cells, especially those in the apical part (**G**, **H**), but not in embryos (H). *SciSox7* is expressed in choanocytes (**I**, **J**) and strongly expressed in the oocytes (J). Top row: oscular parts of young sponges. Bottom row: B - plastic section of sponge containing oocytes. D- thick slice of sponge containing embryos during pre-inversion. F- magnification of the tip of the osculum. H- Plastic sections of sponge containing post-inversion stage embryo. J - thick slice of sponge containing small oocytes.

The expression of *SciSoxE*, *SciSoxF1*, *SciSoxF2*, as well as *SciSox6* was detected in various adult cells and not during embryonic stages or in the larvae (Figure 4). *SciSoxE* expression was detected in choanocytes, but not in the accessory cells [17,19] (choanocyte-derived cells surrounding the oocytes and embryos) (Figure 4A, B). *SciSoxE* was also detected in a fraction of the mesohyl cells. Similarly, *SciSoxF1* was detected in choanocytes; but in contrast to *SciSoxE*, its expression was also detected in the accessory cells (Figure 4C, D). *SciSoxF2* expression was detected in large cells (possibly the myocytes based on the cell shape), which are located in the middle part of the osculum (Figure 4E, F). *SciSox6* expression was detected in all choanocytes, pinacocytes, and in some mesohyl cells near the rim of the osculum of young sponges (Figure 4G, H). *SciSox7* was expressed in the choanocytes of adult sponges (Figure 4I), as well as in the oocytes (Figure 4J) and uniformly in the embryos (data not shown). 

**Figure 5:**
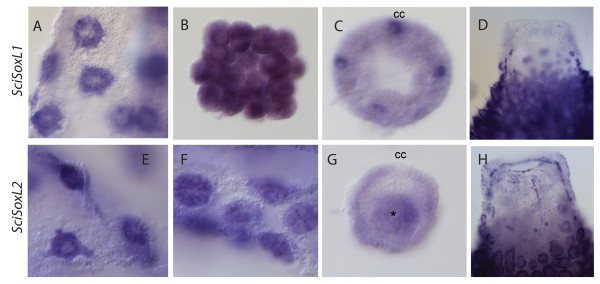
**Expression of *SciSoxL1 *and *SciSoxL2. **** SciSoxL1* is strongly expressed in the oocytes (**A**) and during cleavage (**B**); in pre-inversion stage embryos the expression is limited to cruciform cells (cc) (**C**); the transcripts are also present in the choanocytes (**D**). *SciSoxL2* is strongly expressed in oocytes (**E**) and cleavage stage embryos (**F**); in pre-inversion stage embryos expression in the macromeres (asterisk) is higher than in the micromeres and is undetectable in the cruciform cells (cc) (**G**); the transcripts are also present in choanocytes and a fraction of mesohyle cells (**H**). A and E - thick slices of sponge containing oocytes. B - isolated mid-cleavage stage embryo. F - thick slice of sponge containing cleavage stage embryos. C, G - isolated pre-inversion stage embryos. D, H - oscular parts of young sponges.

Finally, the Sox-like genes *SciSoxL1* and *SciSoxL2* are expressed during embryonic development and in adult cells. *SciSoxL1* is uniformly expressed during early cleavage and during pre-inversion (Figure 5A, B); and then detected in the cruciform cells (Figure 5C). *SciSoxL2* is detected during early embryogenesis (Figure 5E, F) and during pre-inversion in macromeres and weaker in micromeres, but not in the cruciform cells (Figure 5G). *SciSoxL1* and *SciSoxL2* expression was also detected in adult sponges in choanocytes, and *SciSoxL2* also in mesohyl cells in the osculum (Figure 5D-H).

## Discussion

### The Sox gene family is significantly larger in *Sycon* than in *Amphimedon*

As previously reported by Larroux *et al.*[8,9]*Amphimedon* has four Sox genes corresponding to groups B, C, and F. In the demosponge *Ephydatia muelleri* only three Sox genes could be identified [15]. In contrast, the genome of the calcareous sponge *Sycon ciliatum* contains seven Sox genes and four additional Sox-related genes.

In *Sycon*, five Sox genes correspond to the recognized Sox subfamilies, confirming the presence of Sox genes of the groups B, C, and F in sponges, and adding SoxE to the sponge repertoire. While bootstrap support and posterior probabilities values for assigning the poriferan sequences into eumetazoan subfamilies are generally low, analysis of conserved motifs within the full length proteins consistently confirmed placement of the calcaronean sequences within the recognized subfamilies.

There are several differences between the demosponge and calcaronean Sox genes as evidenced by the comparison between *Amphimedon* and *Sycon*. For example, there is only one SoxB gene in *Sycon*. In contrast, the calcaronean sequences can be classified as belonging to SoxE and SoxF families; while only a single (and difficult to place in phylogenetic analysis) SoxF gene is present in the *Amphimedon* genome. The *Amphimedon* SoxE gene might have been lost, or SoxE genes might have evolved after demosponges diverged. It is impossible to differentiate between these two scenarios until the issue of sponge monophyly *vs.* paraphyly is resolved. On the other hand, our result indicates that SoxF genes in *Sycon* and *Leucosolenia* are likely to be a result of lineage-specific duplication.

Interestingly, the *Amphimedon* genome does not appear to contain the large number of Sox-related genes that we have identified in the two calcaronean genomes. It remains unclear whether this is a result of significant gene loss in *Amphimedon*, or rather of expansion of the Sox family in the Calcaronea. Only analysis of additional poriferan genomes representing a range of clades (especially homoscleromorphs, calcineans, and a range of demosponges) will help to shed light into this issue.

### Dynamic expression of Sox genes in *sycon*

The expression patterns of *Sycon* Sox genes fall into two categories: embryonic (*SciSoxB* and *SciSoxC*) or predominantly in differentiated adult cells (*SciSoxE*, *SciSoxF1*, *SciSoxF2*, and *SciSox6*). Sox-like genes are expressed both during development and in adult tissues (Summary on Table 2).

Until functional data are obtained in sponges, the specific roles of the identified genes will remain unclear. However, we can hypothesize on their putative function in *Sycon* and on hypothetical ancestral roles in the metazoan ancestor, by comparing the expression patterns of *Sycon* and the eumetazoan Sox genes. This is particularly tempting for genes belonging to subfamilies that appear to have a conserved function throughout the Eumetazoa, such as the SoxB group. At least one Sox gene belonging to Group B is expressed in the embryonic ectoderm and the neurogenic region of embryos in early development in most bilaterians (for a review see [21]), cnidarians [12,13], and in the ctenophore *P. pileus*[14].

*Sycon* SoxB expression is restricted to two cell types of the embryo, the macromeres and the cruciform cells. During settlement and metamorphosis, the macromeres become the outer cells of the post-larva and subsequently differentiate into exopinacocytes, the outer epithelium of the sponge [22,23]. The *SciSoxB* expression in the macromeres provides support for the notion that the exopinacoderm of the sponges might be homologous to the ectoderm of higher metazoans.

The cruciform cells are characteristic cells of the calcaronean sponge larvae [19,24]. They form from four cytoplasm regions segregated during cleavage and differentiate at the pre-inversion stage; they are present in the swimming larva, to later degenerate during settlement and metamorphosis. Their role is not yet clear, but these four cells are the only candidate cells suggested to play a role in larval photoreception [24]. If the cruciform cells are indeed involved in photoreception, the SoxB expression during their differentiation would indicate conservation of SoxB functions in broadly defined neurogenesis and sensory organ formation [25].

The expression of *Sycon* SoxC is very prominent in macromeres during pre-inversion, while expression was not detected in larvae. In the cnidarians *Acropora* and *Nematostella*, SoxC is expressed during embryogenesis in cell types that are suspected to be sensory neurons [11,12]. However in *Clytia*, SoxC (*ChSox15*) is expressed in stem cells [13]. Therefore it appears that there is no clear conservation of expression pattern among these organisms.

While there is no strong conservation of expression for SoxE and SoxF genes, SoxE genes in bilaterian invertebrates tend to have a role in sex-specific aspects of gonad development, and SoxF genes tend to be associated with endoderm formation [21,26]. In the cnidarians *Nematostella* and *Acropora*, SoxE and SoxF are expressed in endodermal lineages; while in *Clytia* SoxE is expressed in germline cells, stem cells, and nematoblasts [13], indicating once again no clear conservation among cnidarians within this group. However, expression in the endoderm (in Anthozoan cnidarians) and mesodermal derivatives (gonads) of bilaterians, together with the observed expression of *Sycon* SoxE and SoxF in choanocytes and some mesohyl cells, could be used to support a concept of homology of the choanoderm + mesohyl with endomesoderm. Otherwise, these two genes might play roles in cell differentiation in *Sycon*, as evidenced by the fact that expression of SoxE disappears in choanocytes that transdifferentiate into accessory cells, while expression of SoxF1 becomes stronger in these cells during the process.

## Conclusions

Sponges are relatively simple organisms with few cell types, thus the limited number of transcription factors representing conserved metazoan families in the demosponge *Amphimedon quenslandica* fits neatly with the concept of a simple developmental tool kit patterning a simple body. This study demonstrates that *Sycon ciliatum* has multiple Sox genes which are dynamically expressed during development and in patterns consistent with governing adult cell differentiation. This indicates that Sox genes were involved in development and cell differentiation from the beginning of multicellular animal evolution. Further analyses of this and other developmental gene families in the Calcarea and in other sponge group are necessary to test whether the identified differences between *Sycon* and *Amphimedon* are indicative of global differences in the developmental toolkits. Such studies, now underway in our laboratory and in other groups, will provide insight into the evolutionary history of the animal developmental toolkit.

## Methods

### Identification of Sox genes in *Sycon* and *Leucosolenia*

Sox-like genes from *Sycon ciliatum* were retrieved by searching our recently generated genomic and transcriptome databases (Adamski *et al.*, unpublished work) using HMG domain sequences from *Nematostella* and *Amphimedon*. Scaffolds were recovered and annotated using TBLASTN and BLASTX searches. Additionally, we searched in our on-going genome and transcriptome project of another calcaronean, *Leucosolenia complicata*, using the 12 identified *Sycon* HMG domain sequences to recover their orthologs from this species. These sequences were used in the phylogenetic analysis.

*Sycon* Sox genes were amplified by either RACE or RT-PCR using SMART^TM^ RACE Amplification kit (Clontech). Primer sequences are available upon request. The cDNA used as a template was prepared from a mixture of RNA extracted from juveniles and adult samples containing embryonic stages. PCR products were cloned into pGEM-Teasy (Promega) and sequenced using the BigDye Terminator v3.1 protocol (ABI). Purified PCR products obtained using SP6 and T7 primers during colony PCR were used to produce Dig-labeled antisense RNA probes for *in-situ* hybridization (see below).

### Alignment and phylogenetic analysis

Alignment of HMG domains for phylogenetic analyses: MUSCLE [27] was used for the alignment which included *Sycon* and *Leucosolenia* complete HMG domains of candidate Sox genes together with a different combination of taxa (see Additional file 2). The alignment was manually modified where needed. In this final dataset, the following sequences were included: two HMG domains from *Sycon* Tcf genes and out-groups used for phylogenetic analysis as in Jager *et al.*[13]. We did not include the sponge Sox sequences from the previous study in sponges from Jager *et al.*[15] as these HMG domains contain only partial information (59 aa).

Phylogenetic calculations: Prottest 3 [28] was used to determine the best suitable model of protein evolution for our alignment. We used two phylogenetic analyses of HMG domains:

(1)  Two independent runs of PhyML [29] were performed. Each run searched for five random starting trees using SPR moves. The tree with the best log likelihood value was selected (Log likelihood = −5686.2). From this tree a bootstrap analysis using 100 replicates was performed.

(2)  Bayesian analysis [30] under LG model, with 5,000,000 generations sampled every 500 generations using four chains. Convergence was reached before 5,000,000 generations. A majority rule of consensus tree of 12,500 trees was generated and posterior probabilities values were calculated from this tree.

### Finding conserved motifs within sponge Sox sequences

MEME 3.5.7 [31] was used to find conserved motifs outside the HMG domain within *Sycon* and *Leucosolenia* Sox proteins and their closest homologues from *Acropora*, *Homo*, *Nematostella*, and *Amphimedon*. The following parameters were used for searching possible conserved motifs: minimum motif width, six; maximum width, 100; maximum motifs to find, six. Complete sequences were aligned and their motif locations were compared with previous studies [4,12]. ‘My domain image creator’ tool included in Prosite [32] was used to visualize the locations of motifs in Sox proteins.

### Specimen collection and whole mount *in-situ* hybridization

Adult *Sycon* specimens were collected from fjords located near Bergen, Norway (+60° 27' 33", +4° 56' 1") during the reproductive season from May to September (2008 to 2011). For *in-situ* hybridization, samples were immediately fixed in 100 mM MOPS, pH 7.5; 0.5 M sodium chloride; 2 mM MgSO_4_; 4% paraformaldehyde; 0.05% glutaraldehyde over night at 4°C, stepped into and extensively washed in 70% EtOH and stored at −20°C until processing. Macro sections of sponges in 24 well plates (Nunc) were rehydrated and washed in PBS/0.1% Tween (PTw). Samples were pretreated with 7.5 μg/mL proteinase K for 10 minutes at 37°C, followed by quenching with glycine (2 mg/mL PTw). Acetylation was performed by serial treatment with 0.1 M triethanolamine containing 0, 1.5, and 3 μl/mL acetic anhydride. Re-fixation was done in 4% paraformaldehyde/0.05% glutaraldehyde in PBS for 1 h at room temperature, followed by extensive washing in PTw. Tissue was prehybridized as previously described [33] in 2 mL-tubes for 90 to 180 min at 51°C. Probe hybridization was done with denatured RNA probe (0.1-0.3 ng/μL, approximately 1 kb) for 12 to 18 h at 51°C. Stringent washes were carried out at 55°C as following: 1 × 10 min in hybridization buffer; 2 × 10 min 50% formamide/4 × SSC/0.1%; 2 × 10 min 50% formamide/2 × SSC/0.1% Tween; 2 × 10 min 25% formamide/2 × SSC/0.1% Tween, followed by 3 × 15 min 2 × SSC/0.1% Tween at room temperature. Samples were transferred to maleic acid buffer and incubated in 2% (w/v) Blocking Reagent (Roche) for 60 min at room temperature. After overnight incubation with AP-coupled anti-Digoxigenin-Fab fragments (Sigma, 1:5,000) at 4°C, samples were washed in maleic acid buffer at least 6 × 30 min. Probe was detected using NBT/BCIP as substrate (Roche) with tissue equilibrated in alkaline phosphatase buffer (100 mM sodium chloride, 50 mM MgCl2, 100 mM Tris pH 9.5, 0.1% Tween, 1 mM Levamisole). The staining reaction (0.5 to 3 days) was stopped with PBS/0.5% Tween, samples were transferred to 100% glycerol for microscopy or ethanol-dehydrated and embedded in epoxy resin (Sigma) for sectioning. Pictures of whole mount samples and sections were taken using a Nikon DS-U3 microscope and processed in Photoshop.

## Competing interests

The authors declare that they have no competing interests.

## Authors’ contributions

Conceived and designed the study: MajA and SF. Suggested the model system and provided knowledge about its biology: H-TR. Assembled genomes and transcriptomes and created sequence databases: MarA. Carried out sampling and experiments: SF, BB, MarA, CZ, SL, CG, SJ, and MajA. Analyzed data: SF and MajA. Drafted the manuscript: SF. Edited the manuscript: MajA and SF with input from co-authors. All authors read and approved the final manuscript.

## Supplementary Material

Additional file 1 **Maximum likelihood phylogenetic tree of HMG sequences found in*Sycon ciliatum*and*Leucosolenia complicata.*** A phylogenetic analysis which includes the entire repertoire of HMG domains sequences found in *Sycon* (twelve sequences) and *Leucosolenia* (seven sequences). PhyMl tree using LG + G model of protein evolution is shown. Bootstrap support values are displayed. Taxa names: Ami, *Acropora millepora*; Amq, *Amphimedon queenslandica*; Ce, *Caenorhabditis elegans*; Ci, *Ciona intestinalis*; Gdo, *Gallus domesticus*; Hsa, *Homo sapiens*; Lco, *Leucosolenia complicata*; Mm, *Mus musculus*; Ncr, *Neutrospora crassa;* Omy*, Oncorhynchus mykis;* Sci, *Sycon ciliatum*; Xle, *Xenopus laevis*. Click here for file

Additional file 2 **HMG domains recovered from*Sycon*and*Leucosolenia*.** Alignment of *Sycon* and *Leucosolenia* HMG domains of the complete repertoire of sox and sox-like genes recovered for this study. Sequences were compared with: *Acropora millepora* (Ami); and *Amphimedon queenslandica* (Amq). Click here for file

Additional file 3 **Alignment of HMG domains used for the phylogenetic analysis.** Includes the HMG domain sequence alignment used for the phylogenetic analysis in Figure 2. Click here for file

Additional file 4 **Calculation of conserved motifs.** This file includes all taxa used for finding conserved motifs within sponge sequences. *P* values are shown and conserved regions are highlighted in red. Click here for file

## Author details

^1^Sars International Centre for Marine Molecular Biology, Thormøhlensgt. 55, Bergen 5008, Norway. ^2^Department of Biology and Centre for Geobiology, University of Bergen, Thormøhlensgt. 55, Bergen, 5008, Norway.
