# Supplementary figures and images for: Genome-wide analysis of the sox family in the calcareous sponge Sycon ciliatum: multiple genes with unique expression patterns
Source: EvoDevo. 2012 Jul 23;3:14. doi: 10.1186/2041-9139-3-14 (PMC3495037; doi:10.1186/2041-9139-3-14)

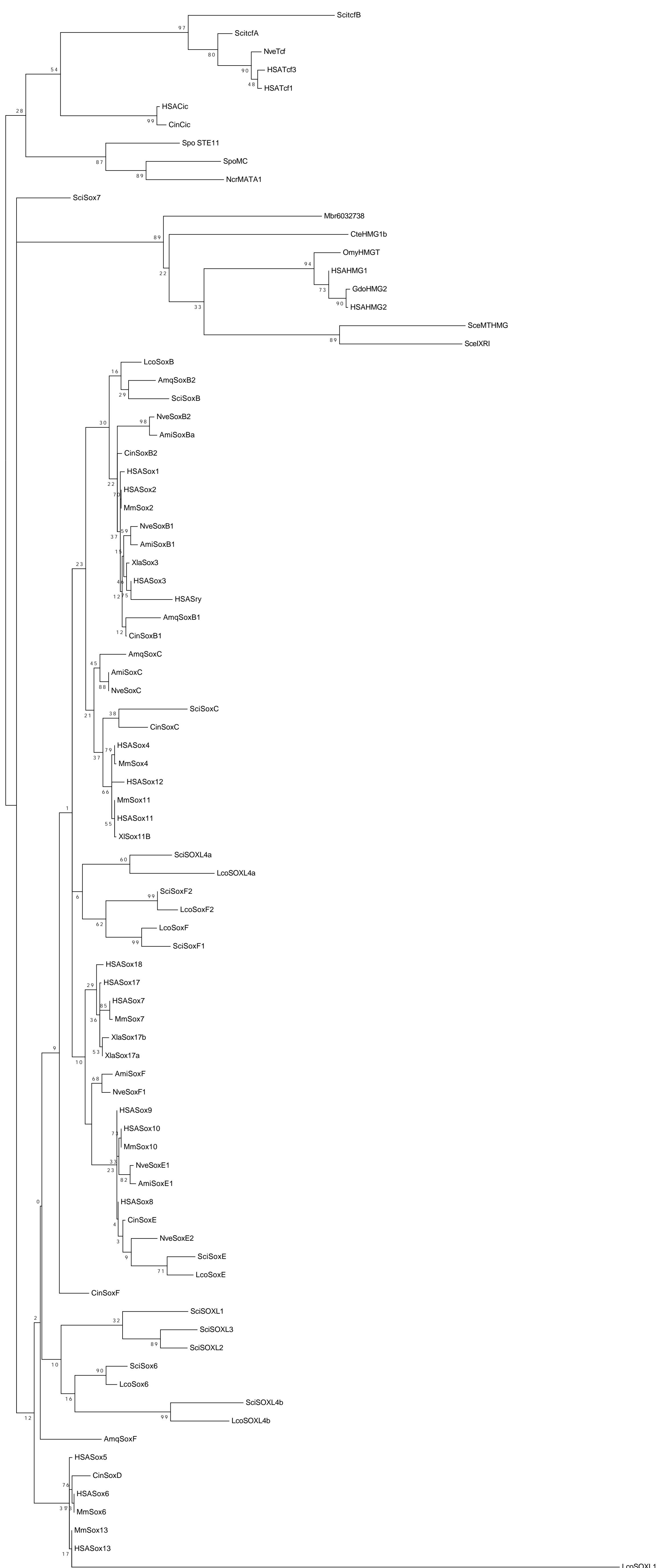

Supplement: Additional file 1 — Maximum likelihood phylogenetic tree of HMG sequences found inSycon ciliatumandLeucosolenia complicata. A phylogenetic analysis which includes the entire repertoire of HMG domains sequences found in Sycon (twelve sequences) and Leucosolenia (seven sequences). PhyMl tree using LG + G model of protein evolution is shown. Bootstrap support values are displayed. Taxa names: Ami, Acropora millepora; Amq, Amphimedon queenslandica; Ce, Caenorhabditis elegans; Ci, Ciona intestinalis; Gdo, Gallus domesticus; Hsa, Homo sapiens; Lco, Leucosolenia complicata; Mm, Mus musculus; Ncr, Neutrospora crassa; Omy, Oncorhynchus mykis; Sci, Sycon ciliatum; Xle, Xenopus laevis. [file 2041-9139-3-14-S1.pdf]
