# Supplementary material for: Genome-wide analysis of the sox family in the calcareous sponge Sycon ciliatum: multiple genes with unique expression patterns
Source: EvoDevo. 2012 Jul 23;3:14. doi: 10.1186/2041-9139-3-14 (PMC3495037; doi:10.1186/2041-9139-3-14)
Supplement: Additional file 2 — HMG domains recovered fromSyconandLeucosolenia. Alignment of Sycon and Leucosolenia HMG domains of the complete repertoire of sox and sox-like genes recovered for this study. Sequences were compared with: Acropora millepora (Ami); and Amphimedon queenslandica (Amq). [file 2041-9139-3-14-S2.pdf]

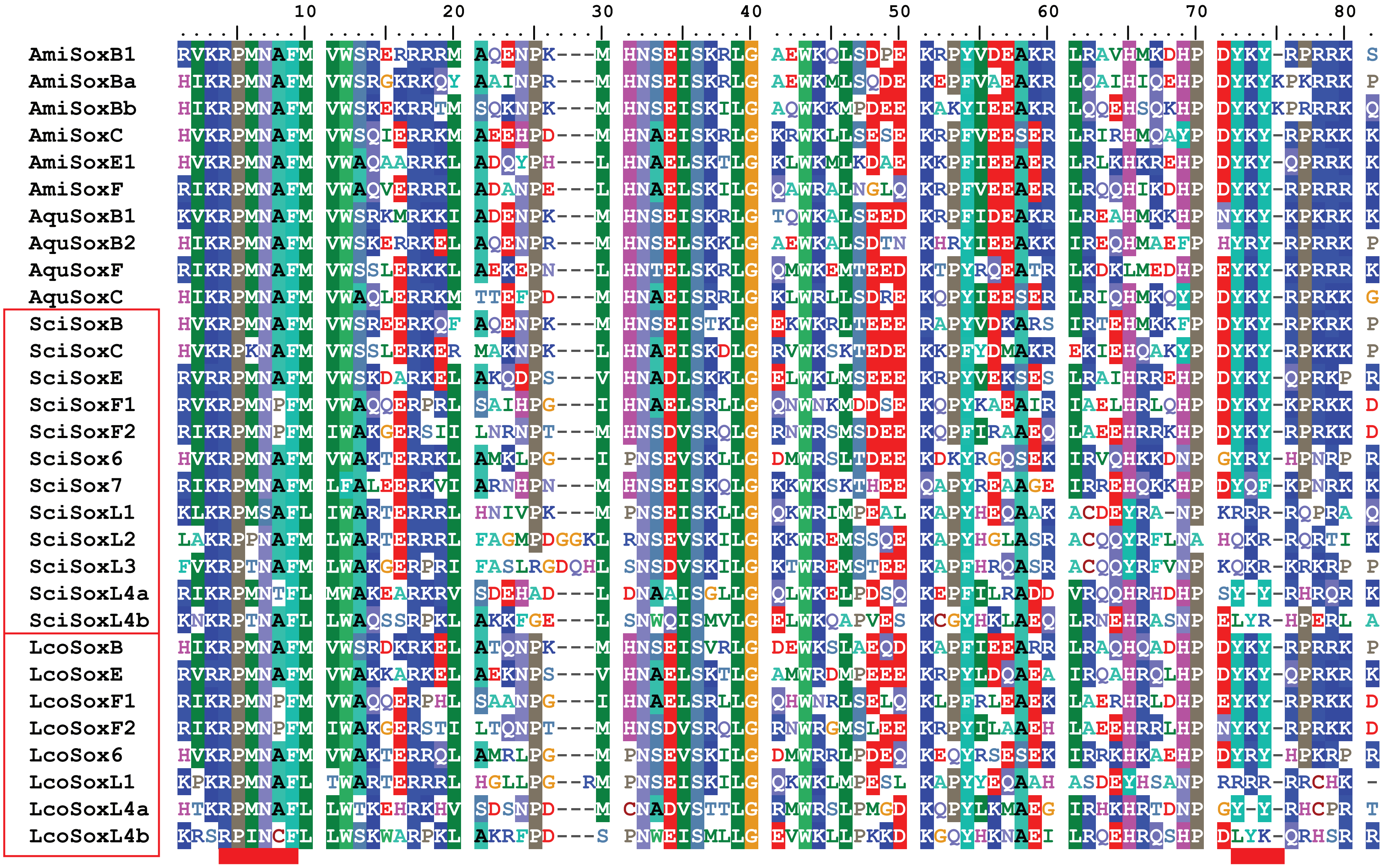

10 20 30 40 50 60 70 80
AmiSoxB1 RVKRPMNAFM VWSRERRRRM AQENPK---M HNSEISKRLG AEWKQLSDPE KRPYVDEAKR LRAVHMKDHP DYKY-RPRRK S
AmiSoxBa HIKRPMNAFM VWSRGKRKQY AAINPR---M HNSEISKRLG AEWKMLSQDE KEPFVAEAKR LQAIHIQEHP DYKYKPKRRK P
AmiSoxBb HIKRPMNAFM VWSKEKRRTM SQKNPK---M HNSEISKILG AQWKKMPDEE KAKYIEEAKR LQQEHSQKHP DYKYKPRRRK Q
AmiSoxC HVKRPMNAFM VWSQIERRKM AEEHPD---M HNAEISKRLG KRWKLLSESE KRPFVEESER LRIRHMQAYP DYKY-RPRKK K
AmiSoxE1 HVKRPMNAFM VWAQAARRKL ADQYPH---L HNAELSKTLG KLWKMLKDAE KKPFIEEAER LRLKHKREHP DYKY-QPRRK K
AmiSoxF RIKRPMNAFM VWAQVERRRL ADANPE---L HNAELSKILG QAWRALNGLQ KRPFVEEAER LRQQHIKDHP DYKY-RPRRR K
AquSoxB1 KVKRPMNAFM VWSRKMRKKI ADENPK---M HNSEISKRLG TQWKALSEED KRPFIDEAKR LREAHMKKHP NYKY-KPRRK K
AquSoxB2 HIKRPMNAFM VWSKERRKEL AQENPR---M HNSELSKKLG AEWKALSDTN KHRYIEEAKK IREQHMAEFP HYRY-RPRRK P
AquSoxF RIKRPMNAFM VWSSLERKKL AEKEPN---L HNTELSKRLG QMWKEMTEED KTPYRQEATR LKDKLMEDHP EYKY-KPRRR K
AquSoxC HIKRPMNAFM VWAQLERRKM TTEFPD---M HNAEISRRLG KLWRLLSDRE KQPYIEESER LRIQHMKQYP DYKY-RPRKK G
SciSoxB HVKRPMNAFM VWSREERKQF AQENPK---M HNSEISTKLG EKWKRLTEEE RAPYVDKARS IRTEHMKKFP DYKY-RPKRK P
SciSoxC HVKRPKNAFM VWSSLERKER MAKNPK---L HNAEISKDLG RVWKSKTEDE KKPFYDMAKR EKIEHQAKYP DYKY-RPKKK P
SciSoxE RVRRPMNAFM VWSKDARKEL AKQDPS---V HNADLSKKLG ELWKLMSEEE KRPYVEKSES LRAIHRREHP DYKY-QPRKP R
SciSoxF1 RVKRPMNPFM VWAQQERPRL SAIHPG---I HNAELSRLLG QNWNKMDDSE KQPYKAEAIR IAELHRLQHP DYKY-KPRKK D
SciSoxF2 RIKRPMNPFM IWAKGERSII LNRNPT---M HNSDVSRQLG RNWRSMSDEE KQPFIRAAEQ LAEEHRRKHP DYKY-RPRKK D
SciSox6 HVKRPMNAFM VWAKTERRKL AMKLPG---I PNSEVSKLLG DMWRSLTDEE KDKYRGQSEK IRVQHKKDNP GYRY-HPNRP R
SciSox7 RIKRPMNAFM LFALEERKVI ARNHPN---M HNSEISKQLG KKWKSKTHEE QAPYREAAGE IRREHQKKHP DYQF-KPNRK K
SciSoxL1 KLKRPMSAFL IWARTERRRL HNIVPK---M PNSEISKLLG QKWRIMPEAL KAPYHEQAAK ACDEYRA-NP KRRR-RQPRA Q
SciSoxL2 LAKRPPNAFM LWARTERRRL FAGMPDGGKL RNSEVSKILG KKWREMSSQE KAPYHGLASR ACQQYRFLNA HQKR-RQRTI K
SciSoxL3 FVKRPTNAFM LWAKGERPRI FASLRGDQHL SNSDVSKILG KTWREMSTEE KAPFHRQASR ACQQYRFVNP KQKR-KRKRP P
SciSoxL4a RIKRPMNTFL MWAKEARKRV SDEHAD---L DNAAISGLLG QLWKELPDSQ KEPFILRADD VRQQHRHDHP SY-Y-RHRQR K
SciSoxL4b KNKRPTNAFL LWAQSSRPKL AKKFGE---L SNWQISMVLG ELWKQAPVES KCGYHKLAEQ LRNEHRASNP ELYR-HPERL A
LcoSoxB HIKRPMNAFM VWSRDKRKEL ATQNPK---M HNSEISVRLG DEWKSLAEQD KAPFIEEARR LRAQHQADHP DYKY-RPRRK P
LcoSoxE RVRRPMNAFM VWAKKARKEL AEKNPS---V HNAELSKTLG AMWRDMPEEE KRPYLDQAEA IRQAHRQLHP DYKY-QPRKR K
LcoSoxF1 RIKRPMNPFM VWAQQERPHL SAANPG---I HNAELSRLLG QHWNRLSELQ KLPFRLEAEK LAERHRLDHP EYKY-RPRKK D
LcoSoxF2 RIKRPMNPFM IWAKGERSTI LTQNPT---M HNSDVSRQLG RNWRGMSLEE KRPYILAAEH LAEEHRRLHP NYKY-RPRKK D
LcoSox6 HVKRPMNAFM VWAKTERRQL AMRLPG---M PNSEVSKILG DMWRRLPDEQ KEQYRSESEK IRRKHKAEHP DYRY-HPKRP R
LcoSoxL1 KPKRPMNAFL TWARTERRRL HGLLPG--RM PNSEISKILG QKWKLMPESL KAPYYEQAAH ASDEYHSANP RRRR-RRCHK -
LcoSoxL4a HTKRPMNAFL LWTKEHRKHV SDSNPD---M CNADVSTTLG RMWRSLPMGD KQPYLKMAEG IRHKHRTDNP GY-Y-RHCPR T
LcoSoxL4b KRSRPINCFL LWSKWARPKL AKRFPD---S PNWEISMLLG EVWKLLPKKD KGQYHKNAEI LRQEHRQSHP DLYK-QRHSR R
