# Supplementary material for: Genome-wide analysis of the sox family in the calcareous sponge Sycon ciliatum: multiple genes with unique expression patterns
Source: EvoDevo. 2012 Jul 23;3:14. doi: 10.1186/2041-9139-3-14 (PMC3495037; doi:10.1186/2041-9139-3-14)

Group SoxB

Group B motif					
**Name**	**Start**	**p-value**	**Sites**		
**HsaSox14**	85	3.61E-13	KYRPRRKPKN	LLKKDRYVFPL	PYLGDTDPLK
**MmSox14**	243	3.61E-13	KYRPRRKPKN	LLKKDRYVFPL	PYLGDTDPLK
**MmSox2**	120	1.22E-11	KYRPRRKTKT	LMKKDKYTLPG	GLLAPGGNSM
**XlaSox3**	117	8.07E-11	KYRPRRKTKT	LLKKDKYSLPG	NLLAPGVSPV
**AmiSoxB1**	108	8.39E-10	YRPRRKSKTL	LKKDNKYTLSM	LGAQGGPPVQ
**CinSoxB2**	90	1.12E-09	YRPRRKPKSL	IKPKDRFAFPL	FPSSGMSSPT
**AmiSoxBb**	87	2.33E-09	YKPRRRKQKQ	LIKKATYSFPY	TGTENAAHAA
**NvSoxB1**	111	4.36E-09	YRPRRKSKTL	LKKDNKYALSM	LSAGQAGGQV
**NvSoxB2**	104	8.89E-08	SPVFSPYAAS	MMAVDKFSTNQ	LPQTIAHSVA
**AmiSoxBa**	104	1.29E-07	GPMFSPYSSP	MMAVDKFPTNQ	LPQTIAHSTA
**CinSoxB1**	174	4.38E-07	YRPRRKTKAI	LKKENKFGMGP	GGIMGGGAGP
**SciSoxB**	199	9.30E-07	NAATIVPAGS	LQPVPVNVFPQ	ANPPAPGRQV
**AmqSoxB2**	249	1.21E-06	DGAKQGGQTI	LIKMDAGTPHQ	VISPSYITAA
**AmqSoxB1**	422	1.30E-06	SSNXTNSAES	LLDGAGGTLPL	QHLM
**LcoSoxB**	254	7.08E-06	FAYMSTAAKP	DIPVAQPTFVM	AANPALPAPG

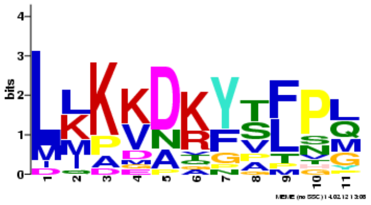

Conserved region I					
**Name**	**Start**	**p-value**	**Sites**		
**HsaSox14**	198	7.28E-09	THPSPTNPGY	VVPCNC	TAWSASTLQP
**MmSox14**	356	7.28E-09	THPSPTNPGY	VVPCNC	TAWSASTLQP
**AmqSoxB1**	336	2.05E-08	PVESLDSYSE	VILSNC	KVSDDCTSIH
**AmiSoxBa**	151	2.79E-07	FHHSVSPGYP	VIYPNV	TAVNSHHSVT
**LcoSoxB**	297	1.47E-06	GHIEQPQVTY	VILPNK	A
**AmqSoxB2**	317	1.72E-06	QSTPTSKTTP	VIVSEI	KSHSTPQVNS
**CinSoxB1**	21	1.91E-06	QIPYPTSGVP	VIPSNA	HLPPPAPIAG
**AmiSoxB1**	233	6.23E-06	PLSYSQPSNQ	VLIPNI	KQEMPSPGSQ
**NvSoxB2**	150	6.67E-06	FHHSVSPGYP	VIYPSV	SNGGNVHSGS
**NvSoxB1**	241	9.08E-06	ITPPYTQHSP	LILPNI	KQEMPSPTGN
**CinSoxB2**	222	1.68E-05	ATTGANKIKS	VLYPNL	LYTFNETEEK
**MmSox2**	244	2.69E-05	PGMALGSMGS	VVKSEA	SSSPPVVTSS
**XlaSox3**	236	8.36E-05	TVMSLASMGS	VVKSEP	SSPPPAITSH
**SciSoxB**	249	1.16E-04	LYTAAPQYQT	FILPSA	APGQPQAVMA
**AmiSoxBb**	173	2.40E-04	ANSVHELPYP	VRPSEM	MIPTPTGPHG

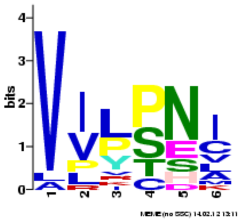

Conserved region II

Name	Start	p-value	Sites		
**MmSox3**	396	1.91E-18	PAIASHSQRA	CLGDLRDMISMYLP	PGGDAADAAS
**XlaSox3**	256	1.91E-18	PAITSHTQRA	CLGDLRDMISMYLP	PGGDASDPSL
**MmSox2**	267	2.11E-14	TSSSHSRAPC	QAGDLRDMISMYLP	GAEVPEPAAP
**NvSoxB1**	266	6.54E-14	NMNVGGVTRN	CADQLGDMINTYLP	PADTANPVST
**AmiSoxB1**	255	1.13E-13	PGSQSSRGRS	CTDQLGDMINTYLP	GESAAASANA
**AmqSoxB1**	357	4.64E-10	CTSIHSNDSG	AESDLRNMISTYLE	ESNSXPGPTE
**CinSoxB2**	274	1.88E-09	HGSRCYEQRL	CDEHTRCWLVVAFP	AKPNPINAES
**HsaSox14**	97	2.11E-08	KKDRYVFPLP	YLGDTDPLKAAGLP	VGASDGLLSA
**MmSox14**	255	2.11E-08	KKDRYVFPLP	YLGDTDPLKAAGLP	VGASDGLLSA
**AmiSoxpB**	98	3.58E-08	KVDRYPFTLP	CLPTTDELLKCAPT	GIPTTSFVTD
**AmqSoxB2**	395	1.77E-07	ALRSAEIPYR	VYPDLSSMPLCLVP	VVDQASPGSN
**LcoSoxB**	180	9.92E-07	VDVRLQRASA	SMPNLHSAQTVYQP	AAPIQHHVVP
**NvSoxB2**	213	1.15E-06	LHSQVQQRLS	QVEDSRGMKSMNAT	PSPPVSSPDP
**CinSoxB1**	243	4.88E-06	PSPHAYPAMA	NSGTAANRYDMYYP	SYSTPTTLPS
**AmiSoxBb**	137	5.23E-06	QQYYQMSQHA	PYPTMYDMAAVHAQ	RQTHSFSTPP
**SciSoxB**	235	1.99E-05	TTQALNGGSS	SATNLYTAAPQYQT	FILPSAAPGQ
**AmiSoxBa**	201	2.26E-05	HNRALYTSQA	FQPTLPSQIQQRIS	SVDEPRGGSL

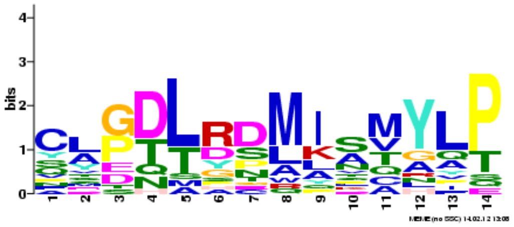

Conserved region III					
**Name**	Start	p-value	Sites		
**XlaSox3**	149	3.74E-17	SSVGVGQRID	TYAHMNGWTNGAYSL	MQDQLGYSQH
**MmSox3**	263	5.57E-16	SPVGVGQRLD	TYTHVNGWANGAYSL	VQEQLGYAQP
**MmSox2**	160	2.59E-12	LGGGLNQRMD	SYAHMNGWSNGSYSM	MQEQLGYPQH
**HsaSox14**	164	2.17E-09	VPHTLATGAL	PYASTLGYQNGAFGS	LSCPSQHTHT
**MmSox14**	322	2.17E-09	VPHTLATSAL	PYASTLGYQNGAFGS	LSCPSQHTHT
**NvSoxB2**	130	5.22E-09	AHSVALSQDP	MYSKINGGPPFHHSV	SPGYPVIYPS
**CinSoxB1**	270	5.26E-08	TPTTLPSMSS	LTSQHNSYAQSAYSV	GGSPAYSVAQ
**AmiSoxB1**	138	1.49E-07	QRSMVQNPAD	HFGQMNGFAYSPITG	YNQMNVNDPY
**AmqSoxB1**	394	1.70E-07	PSGSSRPPTA	EFKLLNASAQCTDFI	ASNSSNXTNS
**AmiSoxBa**	130	2.38E-07	AHSTALSADP	MYSKINGAAAAFHHS	VSPGYPVIYP
**NvSoxB1**	192	7.13E-07	PPMNGLPPHG	SYSNMSASAQIYPSV	SVMTSQPVGA
**LcoSoxB**	148	7.60E-07	TLARAGSATF	SFGSTSEWQSNSSSR	TGSTGSQVDV
**AmiSoxBb**	203	9.75E-07	IYGSTMESGP	TTSGVSAFTNATQNI	HAQQIAEASP
**AmqSoxB2**	157	1.17E-06	PQQVVTSHGL	HHSPIATTTNFIQSL	QPAASIAGGG
**CinSoxB2**	230	1.59E-06	KSVLYPNLLY	TFNETEEKYETHNFR	RRLPMWITTS
**SciSoxB**	151	2.89E-06	TRASSAFNFP	TAMSASGWTSSGTSL	FSSTTPIGQY

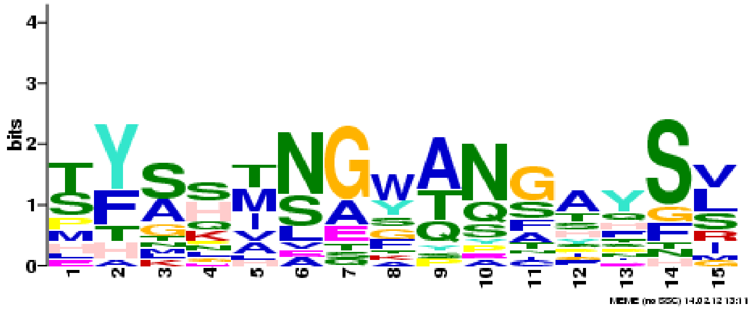

Group SoxC

**Name**	**Start**	**P-value**	**Sites**		
**HsaSox12**	283	1.50E-12	PDLQPPSGTS	HFEFPDYCT	PEVTEMIAGD
**AmiSoxC**	263	3.56E-12	LLDLHGTSAG	QFDFPDLYT	PPEVSELIQG
**CiSoxC**	494	3.26E-10	AYADPCRRKS	HFEFSDYDT	DEVKRMIESS
**AmqSoxC**	368	1.55E-07	FSPFNIDSCS	TFDFPELPS	DFADIFAQNA
**SciSoxC**	554	2.26E-07	EYSCDSNRPL	QFRSPVYYE	SRHTSSMWAP

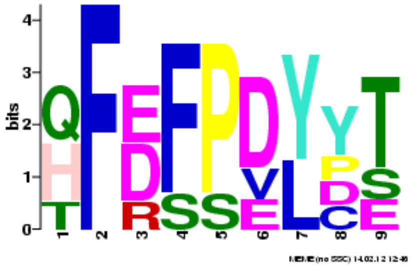

Group SoxE

Conserved motif I

Name	Start	P-value	Sites		
**HsaSox9**	75	2.56E-27	EDKFPVCIRE	AVSQVLKGYDWTLVPMPVRVN	GSSKNKPHVK
**CinSoxE**	177	3.52E-26	KDDMSKDIKD	AVSQVLKGYDWTLVPMPVRMN	GSQKTKPHVK
**MmSox10**	74	7.28E-26	DDKFPVCIRE	AVSQVLSGYDWTLVPMPVRVN	GASKSKPHVK
**NvSoxE1**	34	3.21E-25	NHQLSNAIAS	AVNHVLDGYDWSLIPLPVRVN	GIKTQKPHVK
**AmiSoxE1**	29	3.21E-25	NMDLSSAIAT	AVNHVLDGYDWSLIPLPVRVN	GGHKHKPHVK
**HsaSox8**	70	1.81E-22	DERFPACIRD	AVSQVLKGYDWSLVPMPVRGG	GGGALKAKPH
**LcoSoxE**	345	1.04E-10	DPLTKSFKAG	EWEHVLAAYVSEKCFSPDIFN	TTGFSFSSSA
**SciSoxE**	67	9.03E-10	LTTSPRASPA	PINNEEEQVDAVQKPKNVRKR	KRGDDDRVRR

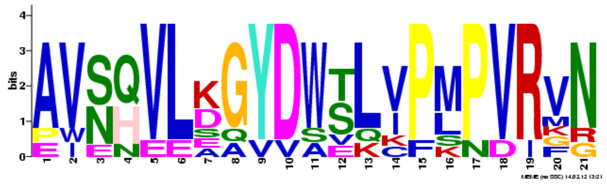

Conserved motif II

Name	Start	P-value	Sites		
**HsaSox9**	266	9.10E-21	RPLPEGGRQP	PIDFRDVDIGELSSDVISNI	ETFDVNEFDQ
**HsaSox8**	257	4.08E-20	GRRPVDSGRQ	NIDFSNVDISELSSEVMGTM	DAFDVHEFDQ
**MmSox10**	269	1.69E-19	GRSLGEGGKP	HIDFGNVDIGEISHEVMSNM	ETFDVTELDQ
**SciSoxE**	233	4.83E-13	TPSLSACGSV	TGGSSDVDVDWLLKDTIQTC	KVSPSMSLNS
**NvSoxE1**	235	2.72E-12	FPSCSKKDDS	NSHAIDFDVGDLTTDLMAMG	DVDSTEFDQY
**CinSoxE**	155	8.99E-11	SSARSASPGT	NDDLSDRDSNPEKDDMSKDI	KDAVSQVLKG
**LcoSoxE**	176	2.14E-09	EDTERLEEPT	AKRFNNTISPALSVDALLQV	QGQYPVDLSR
**AmiSoxE1**	1	6.13E-09	M	TEEATNGEITNTDQRQPKNM	DLSSAIATAV

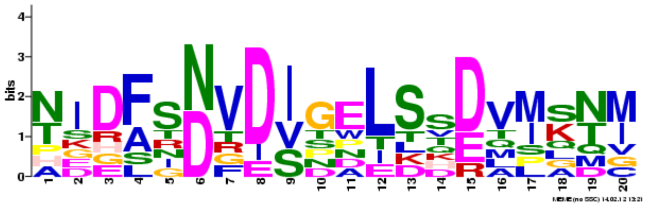

Group SoxF

Name	Start	P-value	Sites		
**HsaSox17**	354	1.19E-19	GTDPSQPAEL	LGEVDRTEFEQYLHFVCK	PEMGLPYQGH
**MmSox17**	359	3.06E-18	GTESNQPTEL	LGEVDRTEFEQYLPFVYK	PEMGLPYQGH
**XSiSox17**	324	3.53E-16	RADHIQQVDM	LAEVDRTEFEQYLSYVAK	SDLGMHYHGQ
**SciSoxF1**	146	1.40E-14	RIKQEEISRV	FKLLSQQEITQYVGLAEK	KRKTKTPKRS
**AmiSoxF**	346	2.29E-14	SSFHITELIP	EEDFNREEFDQYLDGTET	ANISWM
**LcoSoxF1**	142	2.35E-13	RIKQEEISRV	FKSLSQDEITQYVGLASK	KRERPPRRKI
**NvSoxF1**	324	1.60E-12	SIPLSDLLLD	DLNLNTNELDQYLDGTEL	DAFDYVI
**LcoSoxF2**	90	2.65E-11	IDSADQNQRL	FGNNITNEDTQGLPLVRS	KKLVDQASRI
**SciSoxF2**	248	1.10E-10	RLDSDALNRV	TKALTQQEINECVSGCIS	PLMKRAPRPS
**AmqSoxF**	26	1.03E-09	RDREGSEVGE	EENETRNGFQEEEEVVDK	QEEKESKGKK
**CinSoxF**	732	1.12E-09	RKMSVYYDPA	TTAENDAEFAKYEGKATA	AQESVDIKTA

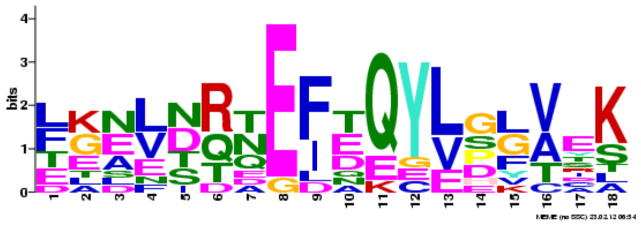

Supplement: Additional file 4 — Calculation of conserved motifs. This file includes all taxa used for finding conserved motifs within sponge sequences. P values are shown and conserved regions are highlighted in red. [file 2041-9139-3-14-S4.pdf]
